# Creatine metabolism differs between mammals and rainbow trout *(Oncorhynchus mykiss)*

**DOI:** 10.1186/2193-1801-3-510

**Published:** 2014-09-09

**Authors:** Andreas Borchel, Marieke Verleih, Alexander Rebl, Carsten Kühn, Tom Goldammer

**Affiliations:** Leibniz-Institut für Nutztierbiologie (FBN), Institut für Genombiologie, Wilhelm-Stahl-Allee 2, Dummerstorf, 18196 Germany; Landesforschungsanstalt für Landwirtschaft und Fischerei Mecklenburg-Vorpommern (LFA M-V), Institut für Fischerei, Born, Germany

**Keywords:** L-arginine:glycine amidinotransferase (GATM), S-adenosylmethionine: guanidinoacetate N-methyltransferase (GAMT), Creatine kinase muscle-type (CKM), Creatine kinase brain-type (CKB), Teleost, Rainbow trout, Energy metabolism

## Abstract

Creatine plays an important role in the cell as an energy buffer. As the energy system is a basic element of the organism it may possibly contribute to differences between rainbow trout strains selected for the traits growth and robustness, respectively. The cDNA sequences of creatine-related genes encoding glycine amidinotransferase (*GATM*), guanidinoacetate N-methyltransferase (*GAMT*), creatine kinase muscle-type (*CKM)* and creatine transporter 1 (CT1, encoded by gene solute carrier family 6, member 8 (*SLC6A8*)) were characterized in rainbow trout. Transcripts of the respective genes were quantified in kidney, liver, brain and skeletal muscle in both trout strains that had been acclimated to different temperatures. Several differences between the compared trout strains were found as well as between temperatures indicating that the energy system may contribute to differences between both strains. In addition to that, the expression data showed clear differences between the creatine system in rainbow trout and mammals, as the spatial distribution of the enzyme-encoding gene expression was clearly different from the patterns described for mammals. In rainbow trout, creatine synthesis seems to take place to a big extent in the skeletal muscle.

**Electronic supplementary material:**

The online version of this article (doi:10.1186/2193-1801-3-510) contains supplementary material, which is available to authorized users.

## Introduction

Products of the fishery industry crucially contribute to world’s nutrition. Since the 1990s the amount of captured fish has been stagnating while the amount of fish produced in aquaculture facilities has been increasing until today (FAO 2012). However, diseases (Meyer 1991) as well as environmental factors like changing seasonal temperatures and concomitant changes in relevant water parameters like oxygen level or pathogen concentration may adversely affect health or even lead to the death of the cultured fish. Such incidents pose a major risk for fish farms and can lead to big economic losses. Therefore, the selection and farming of as robust animals as possible that are adapted to local environments can contribute to sustainable regional aquaculture and ensure a balanced economic efficiency of aquaculture facilities.

The brackish water of the Baltic Sea is challenging regarding pathogens, eutrophication, salinity, temperature and oxygen. A local rainbow trout strain which seems to be robust under and especially adaptable to these fluctuating environmental conditions (Rebl et al. 2012) is the anadromous BORN trout. It has been bred in the brackish water of the Baltic Sea by the Fishery Institute of LFA M-V in the coastal town Born in Germany since 1975 (Anders 1986). Several genes are differentially regulated in BORN trout compared to the typically cultured imported Steelhead trout, which are bred under their native biological conditions, concerning several key aspects like immune system (Köbis et al. 2013; Rebl et al. 2011) or calcium metabolism (Verleih et al. 2012). These differences in gene expression have in part also been shown to be dependent on temperature (Rebl et al. 2013), which is an important abiotic factor or the ‘ecological master factor’ (Brett 1971). This is especially true for poikilothermic animals like fish, as their body temperature is directly correlated to the water temperature. Likewise, temperature influences the growth of pathogens and the outcome of infections (Gilad et al. 2003) and it has a direct impact on the metabolism and hence the oxygen demand (Caulton 1977).

An important molecule affecting the homeostasis of the energy budget and the complete cellular metabolism is creatine (Wyss & Kaddurah-Daouk 2000). In combination with its phosphorylated form, creatine acts as an energy buffer and also allows the energy transport between different cell components as well as organs. Creatine phosphate is used for the regeneration of ADP to ATP by providing the necessary phosphate groups, thereby maintaining an adequate ATP level. As creatine is an energy-buffer, it can mainly be found in tissues with a high energy demand and a high energy flux. Highest levels can therefore be found in skeletal muscle as well as spermatozoa and also the brain in mammals. Up to 94% of the total creatine content can be found in the muscles (Wyss & Kaddurah-Daouk 2000), whereas the basal total creatine concentration is low in kidney and liver (Ipsiroglu et al. 2001).

**Figure 1:**
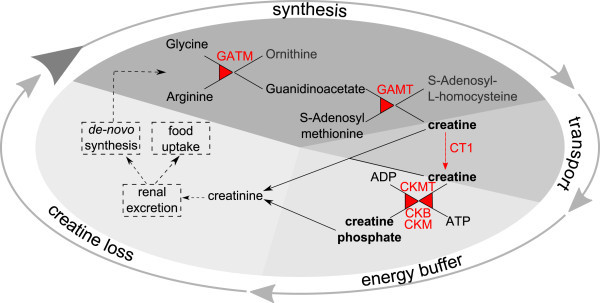
**Schematic overview over the creatine pathway.** The synthesis of creatine is a two-step mechanism (Wyss & Kaddurah-Daouk 2000). In the first step GATM produces guanidinoacetate and ornithine based on glycine and arginine. Guanidinoacetate is subsequently converted to creatine by GAMT. Finally, creatine phosphate is generated by creatine kinases like CKMT, CKB or CKM, which use the phosphate groups of ATP molecules to phosphorylate creatine molecules. They also catalyse the reverse reaction, the phosphorylation of ADP by creatine phosphate. The creatine transporter CT1 is in charge of the transport of creatine through the membranes of the target cells. As around 2% of the total creatine content is non-enzymatically converted to creatinine per day, which is excreted, creatine has to be synthesized continuously or to be taken up by diet.

Creatine can be obtained exogenously from nutrition or it can be synthesized intrinsically. The synthesis of creatine is a two-step mechanism (Figure 1), involving the enzymes glycine amidinotransferase (GATM alias AGAT) and guanidinoacetate N-methyltransferase (GAMT). Sodium- and chloride-dependent creatine transporter 1 (CT1, encoded by gene solute carrier family 6, member 8 (*SLC6A8*)) is in charge of the transport of creatine through the membranes of the target cells. Phosphorylation and dephosphorylation of creatine is performed by creatine kinases (CKs) of brain-type (CKB) or muscle-type (CKM) as well as mitochondrial creatine kinases (CKMT). CKMT directly phosphorylate creatine in the mitochondria, whereas the converse reaction is performed by the cytosolic kinases CKB and CKM (Fritz-Wolf et al. 1996). In humans, deficiency of one of the enzymes of the creatine pathway leads to severe health-related problems, summarized as cerebral creatine deficiency syndrome (CCDS) including intellectual disability, slowed development and epilepsy (Mercimek-Mahmutoglu et al. 2009).

The importance of the creatine system for fish has not been focused so far. Nevertheless it was shown in 1929 that fish muscles have a higher creatine content than mammalian muscles (Hunter 1929) indicating a high relevance. Additionally it was shown that creatine supplementation leads to higher endurance in a fixed velocity test in rainbow trout (McFarlane et al. 2001). Considering zebrafish, the tissue distribution of GATM, GAMT and CT1 is comparable with humans (Wang et al. 2010).

This manuscript investigates the creatine system of two rainbow trout strains, the locally adapted strain BORN and an import strain. Therefore we isolated and characterized the open reading frames (ORFs) of *GAMT*, *GATM*, *CKM* and a fragment of *SLC6A8* including the quantification in both trout strains at different temperatures and in different tissues. To examine the effect of temperature upon the creatine system, we used a temperature challenge experiment and compared gene expression of *GAMT*, *GATM*, *CKM*, *CKB* and *SLC6A8* in kidney, liver, brain and muscle, as these organs are known to be important in the mammalian creatine system.

## Materials and methods

### Experimental animals, temperature challenge and sampling

Rainbow trout of strain BORN and import strain were grown at the same time from eyed eggs to fingerlings under similar conditions in fresh water, followed by an adaptation to fresh water glass tanks at the age of 7–8 month. 10-month old rainbow trout of both strains were used for the experiment. Ten fish per strain were transferred into two separate 300-l freshwater tanks and adapted to 15°C for two weeks. After this first acclimation, the water temperature was gradually adjusted by 1°C per day until respective temperatures of 8°C and 23°C were reached. The fish were kept at these temperatures for one week and were then sacrificed with an overdose of benzocaine. The fish were dissected and kidney, liver, skeletal muscle and brain tissue were obtained from all fish and stored in RNAlater (25 mM Na_3_C_6_H_5_O_7_; 9.9 mM EDTA; 5.3 M (NH_4_)_2_SO_4_) at -80°C until further use. These organs were chosen as they are known to be important in the mammalian creatine system. DNA was isolated from kidney tissue using the QIAamp DNA Micro Kit (Qiagen, Hilden, Germany), in order to determine the gender. While the Import strain is a completely female strain, BORN trout sampled at 8°C comprised seven females and three males; BORN trout acclimated to 23°C included six males and four females.

### RNA extraction & cDNA synthesis

Flash-frozen animal tissues were homogenized in 1 ml Trizol (Invitrogen, Karlsruhe, Germany). RNA extraction was performed using the RNeasy Mini Kit (Qiagen, Hilden, Germany). On-column DNase treatment of the samples ensured the absence of genomic DNA. RNA integrity was verified by agarose gel electrophoresis and quantity was measured using a NanoDrop ND-1000 spectrophotometer (NanoDrop Technologies, Wilmington, DE, USA). On average, 260/280 as well as 260/230 ratios were larger than 2, indicating high quality RNA. 1.5 μg of the RNA were then deployed in cDNA synthesis using Superscript II (Invitrogen) as reverse transcriptase and Oligo-d(T)_24_-primers. The cDNA was treated with the High Pure PCR Product Purification Kit (Roche, Mannheim, Germany) to purify the nucleotides and diluted in 100 μl nuclease free water.

### Isolation of *GATM*, *GAMT*, *SLC6A8*and *CKM*

Unlike *CKB*, *GATM*, *GAMT, SLC6A8* and *CKM* have not been isolated in rainbow trout so far. BLAST algorithm (Altschul et al. 1990) was used to determine gene-specific ESTs in the database of the Gene Index Project (http://compbio.dfci.harvard.edu/tgi/) based on corresponding sequences of other teleost’s like salmon or Japanese rice fish. Then, gene-specific primers flanking the complete coding region were deduced. All primers used in this study are listed in Table 1. PCR was carried out using HotStarTaq Plus DNA polymerase (Qiagen). After a five minute activation at 95°C, 35 cycles were performed including 30 seconds denaturation at 94°C, 30 seconds annealing at 60°C and 90 seconds elongation at 72°C, completed by a five minute final elongation step at 72°C. Resulting PCR-products were cloned into pGEM-T Easy (Promega, LaJolla, CA, USA), if necessary and sequenced for at least three times. Sequences were translated using the virtual ribosome (Wernersson 2006). Sequence comparison was performed using ClustalW (Thompson et al. 1994) and the distance matrix function of UGENE (Okonechnikov et al. 2012). Conserved domains were identified using CD-Search (Marchler-Bauer & Bryant 2004) and the probability of protein export to mitochondria was calculated using MitoProt II (Claros & Vincens 1996).

**Table 1:** **Primers used in this study**

Primer name	Source sequence	Sequence (5′–3′)
*Amplification of coding sequences from rainbow trout*		
OM_GATM_CDS_f	BX868137	CCGCCGCTAGAATATCCCAAAT
OM_GATM_CDS_r	DV201821	TGCAGATTGTTGATTGGGACTTT
OM_GAMT_CDS_f	CR374244	AGACAGCAACTCCGTCCATC
OM_GAMT_CDS_r	BX076691	GCACTTGAGAAGGCATGACA
OM_CKM_CDS_f	CT569995	GGCTCTGGTGAACAGGATCTGA
OM_CKM_CDS_r	CT569258	GGTTGGCTCAATGGCACATAAC
*Amplification of SLC6A8-fragment from rainbow trout* ^*a*^		
OM_SLC6A8_frag_f	cf. methods	CCTCCATGGTGATTGTSTTCT
OM_SLC6A8_frag_r	cf. methods	CRCTGGCWGAGTAGTAGTCAAA
*Quantification of transcripts*		
OM_GATM_qPCR_f	**HG315738**	ACCTCTACTGGCATGTATGCTG
OM_GATM_qPCR_r	**HG315738**	CTTGGCACCCTTTCTGAAGTAC
OM_GAMT_qPCR_f	**HG315739**	TCGACAACATGTTCCAGGAGAC
OM_GAMT_qPCR_r	**HG315739**	GCAGTGGCATCAAGCCATTTCA
OM_CKB_qPCR_f	FJ548753	ATAACCCAGGCCACCCCTTCA
OM_CKB_qPCR_r	FJ548753	TGGGTTCAGGTCGGTCTTGTG
OM_CKM_qPCR_f	**HG315740**	TGCGTTGGTCTGAAAAGGATTGA
OM_CKM_qPCR_r	**HG315740**	TCTCCTCAAACTTGGGGTGTGT
OM_SLC6A8_qPCR_f	**HG315741**	GGAAGCCCAGGTGTGGATTGA
OM_SLC6A8_qPCR_r	**HG315741**	AAAGAAACTGGTCCCACTGTTGA
OM_EEf1A1_qPCR_f	NM_001124339	TGATCTACAAGTGCGGAGGCA
OM_EEf1A1_qPCR_r	NM_001124339	CAGCACCCAGGCATACTTGAA
OM_RPS5_qPCR_f	NM_001160519	ATGACATCTCACTGCAGGATTAC
OM_RPS5_qPCR_r	NM_001160519	ATCAGCTTCTTGCCGTTGTTGC

^a^Degenerated primers.

Sequences obtained in this study are printed bold.

As no EST containing the creatine transporter gene *SLC6A8* of rainbow trout was available, a different approach was used for this gene. Degenerated primers were derived from evolutionarily conserved sequence regions of other closely related teleost species using the Primaclade software (Gadberry et al. 2005). The *SLC6A8*-sequence of the zebrafish *Danio rerio (ENSDART00000037922)* was obtained from ENSEMBL and was aligned with the fitting sequences from *Tetraodon nigroviridis (ENSTNIT00000009059)*, *Takifugu rubripes (ENSTRUT00000032470)* and *Oryzias latipes* (ENSORLT00000023266) using ClustalW (Thompson et al. 1994). Two primers suggested by Primaclade were used to generate a 1134-bp long fragment that was cloned into pGEM-T Easy and sequenced.

### Transcript quantification

Semiquantitative PCR was performed including 5 minutes of initial denaturation at 95°C followed by 30 (*GAMT*, *CKB*, *CKM*) or 35 (*GATM*, *SLC6A8*) cycles of 30 seconds denaturation at 94°C, followed by 30 seconds annealing at 60°C, and 20 seconds elongation at 72°C. PCR was finished with a final 5-minute elongation step at 72°C. Primers were deduced from the trout sequences using the PSQ Assay Design software (Biotage, Uppsala, Sweden). *EEF1A1* was used as a reference gene in parallel and was applied together with the other PCR products onto 2.5% agarose gels containing ethidium bromide, enabling visualization under UV-light. For this first experiment one fish of the import line that had been acclimated to 8°C was used. Band intensities were quantified densitometrically with the tool ImageJ (Schneider et al. 2012).

Transcript quantification was performed using quantitative real-time PCR on a LightCycler 480 system (Roche) and the SensiFast SYBR No-ROX Kit (Bioline, London, UK). 5 μl of cDNA were used per assay. As qRT-PCR program, we used an initial activation step of 5 min at 95°C, followed by 40 cycles of 15 s denaturation at 95°C, 10 s annealing at 60°C, 20 s elongation at 72°C and final quantification for 5 s at 75°C. Product size and quality of the resulting PCR products were visualized through separation in 3% agarose gels. The copy numbers for each gene were calculated based on specific external standards and normalized with the geometric mean of the expression of the reference genes *EEF1A1* and *RPS5*. Significance levels of observed differences were calculated using t-tests, considering p-values < 0.05 significant.

## Results

### Isolation and characterization of *GATM*, *GAMT*and *CKM*

GATM, GAMT, CKB and CKM are enzymes relevant in creatine metabolism. As only the gene encoding CKB has been identified in rainbow trout so far, we isolated *GATM*, *GAMT* and *CKM*.

**Figure 2:**
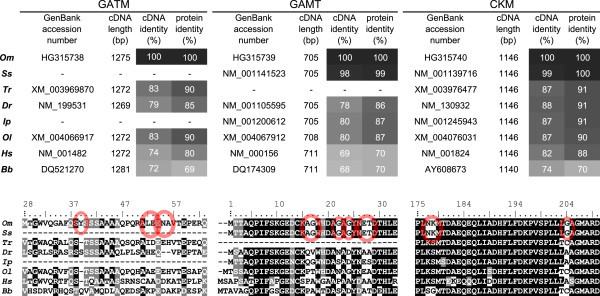
**Sequence comparisons between creatine-related genes of trout and other species.** cDNA sequences encoding the ORFs of *GATM*, *GAMT* and *CKM* were compared between *Oncorhynchus mykiss* (*Om*), *Salmo salar* (*Ss*), *Takifugu rubripes* (*Tr*), *Danio rerio* (*Dr*), *Ictalurus punctatus* (*Ip*), *Oryzias latipes* (*Ol*), *Homo sapiens* (*Hs*) and *Branchiostoma belcheri tsingtauense* (*Bb*). GenBank accession numbers are given in each first column. The figure’s upper part gives an overview over cDNA length, cDNA identity after alignment and protein identity after translation and alignment in relation to trout sequences. Values are shown for GATM (left), GAMT (middle) and CKM (right). High identity levels have a dark background, lower levels a lighter one. Below, the corresponding multiple sequence alignments are shown on protein level. Such regions are shown, that allow a discrimination of the salmonid protein from the proteins of other species. Respective amino acids are encircled. Conserved, identical amino acids are shaded black, similar ones grey. The ruler gives the amino acid position on the trout protein sequence.

The open reading frame of *GATM* was longest with 1275 bp (accession number HG315738), followed by *CKM* (1146 bp, HG315740) and *GAMT* (705 bp, HG315739). Complete multiple sequence alignments of the corresponding protein sequences and of orthologues of other species are given in Additional file 1. A summary is given in Figure 2.

Rainbow trout’s GATM was similar to the GATM of other fishes (up to 90% protein identity) and humans (80% protein identity) but had fewer matches with the sequence of Belcher’s lancelet (*Branchiostoma belcheri,* 69% protein identity). Compared with the sequences of other fishes, trout’s GATM showed an insertion of one amino acid at position 39 (tyrosine). While the complete alignment showed an overall very high conservation, the sequences were quite diverse between position 30 and 65 of the alignment. The probability of export to mitochondria for trout’s GATM was calculated as 97%.

For GAMT the protein sequence was to 99% identical with the sequence of salmon, showing only one amino acid exchange, while on mRNA level 10 base exchanges were observed (not shown). The protein sequence showed also a high accordance with the sequences of the other regarded fishes (≥86% protein identity) but also with human (70% identity) and even lancelet (70% protein identity). The amino acids recognized as S-adenosylmethionine binding sites seemed to be very strongly conserved.

Also the *CKM* cDNA from trout encoded for the identical protein as the one that has been found in salmon. The protein sequence is 100% identical in spite of 12 base exchanges on mRNA level. All recognized ADP-binding sites as well as creatine-binding sites were completely conserved between the examined species. In the substrate specificity loop 16 out of 20 amino acids were found to be completely conserved. Very strong conservation was observed inside the vertebrate group with a protein identity of 88% between trout and human. Only the lancelet had a comparatively low identity of 70%.

The sequence information obtained, enabled us to deduce primers for PCR and to quantitate the respective transcripts.

### Tissue distribution *via*semiquantitative PCR

**Figure 3:**
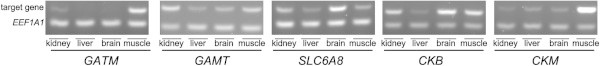
**Representative gels of semiquantitative PCR of five transcript fragments encoding factors of the creatine pathway.** Expression of *GATM*, *GAMT*, *SLC6A8*, *CKB* and *CKM* in kidney, liver, brain and muscle was studied using semiquantitative PCR. *EEF1A1* was used as a reference gene. The gel photos show results of one import trout. The cycle number was higher for *GATM* and *SLC6A8* (35 cycles) than for the other genes (30 cycles). The lower bands represent *EEF1A1* and the upper bands the target genes.

As kidney and liver are the main organs of creatine synthesis and brain and muscle are the main organs of creatine usage in mammals, we decided to have a look at the gene expression of the genes involved in creatine metabolism in these tissues. At first, semiquantitative PCR was used to get a general overview over the tissue distribution. In fact, the expression of these genes was tissue-specific (Figure 3), while the expression of *EEF1A1* that was used as reference gene was constant between the regarded tissues (densitometric analysis: intensities between 36000 and 40000). A very prominent *GATM* band (intensity 31701) was observed after PCR of muscular cDNA, while the band of kidney was plainly fainter (8322). In liver as well as in brain no *GATM* band appeared. Considering *GAMT*, the strongest bands were obtained in kidney and muscle (34993 and 26444), while the bands of liver and brain were less intense (9323 and 15735). *SLC6A8* had its maximum in the brain (30474) but was found in the other tissues as well. *CKB* was found in great amounts in the brain (29834), the muscle (22392) and to a lesser extent the kidney (18587) and very little in the liver (3218). *CKM* showed a very clear maximum in the muscle (39472) and showed only faint bands in the other tissues (4400–5200).

### Tissue distribution *via*qRT-PCR

**Figure 4:**
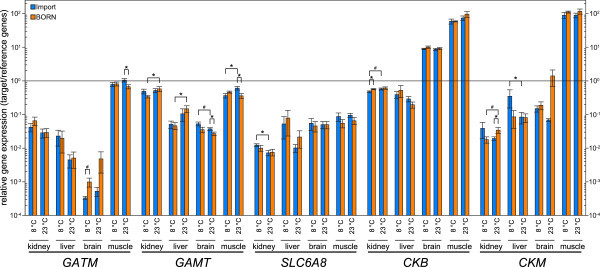
**Expression profiles of five genes encoding factors of the creatine pathway in four tissues.** Relative copy number of *GATM*, *GAMT*, *SLC6A8*, *CKB* and *CKM* was measured in kidney, liver, brain and muscle (8 fish per condition) in relation to the reference genes *EEF1A1* and *RPS5* using qRT-PCR. Import and BORN trout had been acclimated to 8 and 23°C. Values are shown on a logarithmic scale and are means ± SEM. The line indicates an as high expression of the target gene as the expression of the reference genes. Significance levels between strains and temperatures are marked with an asterisk (*, p < 0.05) and hash sign (#, p < 0.1).

Quantitative RT-PCR confirmed the findings of the semiquantitative PCR for *GATM*, *GAMT*, *CKB* and *CKM*. Only SLC6A8 showed a slight difference (Figure 4). Comparatively high expression of *GATM* was found in muscle (average relative expression 0.67-1.05) being up to 35 times as high as expression in kidney, which was the organ with the second highest *GATM*-expression (0.03-0.07). In contrast to that, *GATM* expression in brain was almost negligible (0.0003-0.004). The expression of the gene *GAMT* was high in kidney (0.34-0.58) and muscle (0.36-0.61) and reached only one tenth of these values in liver (0.05-0.15) and brain (0.03-0.05). Creatine transporter gene *SLC6A8* showed quite comparable expression levels in liver, brain and muscle (0.01-0.09), whereas the expression in kidney was lower (0.007-0.01).

Both examined creatine kinases, *CKB* and *CKM*, were expressed strongest in muscle. While a quite high basal expression of *CKB* could be detected in all examined tissues, *CKM* expression seemed more tissue-specific showing a high expression almost exclusively in muscle (89–116), reaching the highest expression values measured in this experiment. While *CKB* showed a high expression in brain (9–10) as well as muscle (59–96), a strikingly high expression for *CKM* was only observed in muscle being 90–100 times as high as the expression of the reference genes. In kidney and liver expression of *CKB* was stronger than expression of *CKM*.

### Differences between trout strains and acclimation temperatures

In addition to the distribution pattern of creatine-related genes, we found several significant differences in their expression between both trout strains and between acclimation temperatures. Animals of strain BORN that were acclimated to 23°C showed decreased *GAMT*-expression in brain (Fold-change (FC) = -1.4; p = 0.047) and muscle (FC = -1.7; p = 0.033) in comparison to the import strain. In addition to that, BORN trout showed an up-regulation of the *GAMT* expression at 23°C in kidney (FC = 1.7; p = 0.041) and liver (FC = 3.17; p = 0.031). Import trout acclimated at 8°C showed a significant lower *GAMT* expression in the muscle in comparison to 23°C acclimated animals (FC = -0.6; p = 0.032).

Further strain-specific differences were found for *GATM* in the muscles of 23°C acclimated animals, for *CKB* in kidneys of 8°C acclimated animals, and for *CKM* in the kidneys of 23°C acclimated animals. Additional significant effects of acclimation temperature could be observed for *SLC6A8* as well as *CKB* in the kidney of import trout, and for *CKM* in the liver of import trout.

Considering the temperature dependence of the gene expression, most genes were regulated in the same way in most tissues. Only in muscle, BORN and import trout showed a different regulation of their creatine metabolism related genes at 8 or 23°C, respectively. While *GATM* and *GAMT* expression were higher in muscle at 23°C than at 8°C in import trout, it was the other way around for BORN trout. For *SLC6A8* and *CKM* the pattern was opposite. In addition to that, *CKM* was differentially regulated in all tissues except the liver. In kidney, brain and muscle BORN trout had a higher gene expression of *CKM* at 23°C, whereas import trout showed higher expression at 8°C.

## Discussion

In mammals, there is a quite strong spatial separation of the different steps of creatine synthesis and consumption. Guanidinoacetate (GAA) is produced by GATM in the kidney, then converted to creatine by GAMT in the liver and finally transported to the consumer tissues via a transporter (Wyss & Kaddurah-Daouk 2000). Surprisingly, we did not find a comparable tissue distribution of the expression of genes involved in creatine metabolism, despite the very strong sequence conservation among vertebrates including fish. Instead, the highest expression of the two genes encoding enzymes involved in creatine synthesis was observed in muscle, in which also the highest gene expression of creatine kinases was found. This indicates that the muscle is independent of the import of creatine at least to a certain extent. Instead, it seems to produce creatine by itself, contradicting findings in mammals that there is no or only negligible synthesis of creatine in muscle (Wyss & Kaddurah-Daouk 2000; McFarlane et al. 2001; Lee et al. 1994). One reason for these differences may be the different creatine amounts in the muscle. Fish muscles have higher creatine contents than mammalian muscles (Hunter 1929). It may be energetically beneficial to maintain such a high creatine level directly at the place of usage instead of transporting it through various organs. Reason for the different creatine levels might be the generally different locomotor activity of fish and mammals. In rat skeletal muscle, only 10% of GATM activity of the according rat liver was observed (Daly 1985). Nevertheless, there are some studies indicating a more important role of muscular creatine synthesis than generally assumed. Schmidt et al. (Schmidt et al. 2004) found strong expression of *GAMT* mRNA and protein in skeletal muscle of humans and found a similar pattern in mice. Also in humans, deGrauw (deGrauw et al. 2003) and his colleagues found significant amounts of creatine in the skeletal muscle of a patient with a creatine transporter deficiency, which may also indicate creatine synthesis in muscle. Finally, also McClure concluded from his studies with mice that ‘*de novo* creatine synthesis can occur in skeletal muscles of mature *mdx* mouse’ (McClure et al. 2007).

However, we found an expression of the gene encoding the creatine transporter *SLC6A8* being as high in muscle as in the other tissues we examined, indicating that an import also takes place. It is also possible, that the expression of *GATM*, *GAMT* and *SLC6A8* may be specific for different cell-types in trout’s muscle. In rat’s brain it was observed that different cell types showed different expression patterns of creatine-related genes (Braissant & Henry 2008). Different cells expressed different combinations of the three genes *GATM*, *GAMT* and *SLC6A8*, reaching from no expression at all over the expression of one or two genes, up to the expression of the complete set of these genes. It was supposed that a transport of creatine between these different cells may still be necessary and therefore a creatine transporter is needed. By and large, muscle seems not only to be an important user of creatine, but also to be a major organ of creatine production in rainbow trout. This should be confirmed on protein level in further examinations.

Although the theory of spatially distributed creatine production and consumption is quite old, newer studies revealed that in mammals the supply with creatine for the brain is not totally dependent on import processes. In addition, there is also a creatine production in the central nervous system (CNS) itself (Béard & Braissant 2010). The discussion about the importance of creatine import into the CNS is controversial. On the one hand the creatine transporter CT1 might be a ‘major pathway to the brain’ (Ohtsuki et al. 2002) for creatine via the blood–brain barrier. On the other hand there might be ‘a limited permeability’ (Braissant et al. 2010) of the blood–brain barrier for creatine due to missing *SLC6A8* expression in astrocytes attached to microcapillary endothelial cells.

A recent review states that creatine is taken up through the blood–brain barrier in limited amounts, but that the CNS remains dependent on endogenous synthesis (Braissant 2012). We found a strong expression of creatine kinases in the brain of rainbow trout, indicating an expectedly strong energy demand and also an average expression of *SLC6A8* as well as *GAMT*. Only *GATM* showed a considerably lower expression in comparison to its expression in other tissues as well as in comparison to the expression of the other creatine-related genes in the brain. As the formation of GAA is the rate-limiting step of creatine synthesis (Sandell et al. 2003; Wyss & Wallimann 1994), this finding is quite unexpected. One possible explanation is that the creatine transporter CT1 not only transports creatine to the brain but is also capable of transporting the precursor GAA as it has been described elsewhere (Tachikawa et al. 2009). In this case, the main function of CT1 would be the transport of GAA into the brain, where it then is metabolized to creatine by the abundant GAMT.

All examined genes were expressed in liver and kidney. In contrast to findings in mammals, where *GAMT* expression is highest in liver, its expression in rainbow trout was higher in kidney. Interestingly, renal *GAMT* expression was even higher than that of *GATM* which was shown to have a very strong and almost exclusive expression in the kidney of mammals.

There are not many examinations of the piscine creatine system yet. To our knowledge, studies on the distribution of *GATM*, *GAMT* and *SLC6A8* have only been conducted in the zebrafish *D. rerio* (Wang et al. 2010; Wang et al. 2007), where quite different results were observed. In the examined tissues, *GATM* was expressed strongest in the brain, but absent from liver. Expression of *GAMT* was very strong in the heart and also in the liver but almost absent from brain. *SLC6A8* expression was marginal in the liver but was most abundant in brain (Wang et al. 2010). Regarding the creatine system, rainbow trout seems to be rather different from zebrafish showing a broader gene expression of all creatine-related genes in all tissues. However, the evolutionary distance between rainbow trout and zebrafish is quite large. The last common ancestor lived around 250 million years ago (Betancur-R et al. 2013). This could explain differences between both creatine systems. As muscle was not examined in the studies on zebrafish (Wang et al. 2010; Wang et al. 2007), it remains unclear if the strong muscular expression of creatine-related genes is a characteristic of the species rainbow trout alone or if it is typical for fish in general. Therefore, further studies have to be performed in the group of fish to get a broader view of the piscine creatine system.

Several significant differences between BORN and import trout have been observed. They did not deliver a really clear image, as differences were in part contradicting to each other and were also dependent on temperature. Nevertheless, these findings indicate a somehow differential creatine system between BORN and import trout. As the creatine system is a very important energy system this suggests energetic differences between BORN and import trout. This may be either the conclusion of or the reason for some of the differences, which have been found between both strains of rainbow trout yet. A different energy budget may influence the immune system, as the maintenance of this system is rather energy intensive and always is a trade-off between immunity and other energy-demanding processes like growth (Lochmiller & Deerenberg 2000). Furthermore, the synthesis of creatine is expensive as creatine synthesis accounts for 40% of the methyl groups of S-adenosylmethionine and uses 20–30% of the amidino groups of arginine (Brosnan et al. 2011). This underlines the meaning of creatine synthesis in amino acid metabolism. Further research on this field may lead to the disclosure of the reasons of the differences between BORN and import trout.

In addition to the difference between both trout lines, differences between the acclimation temperatures of the fish (8°C; 23°C) were observed. A certain effect of temperature on the expression of creatine-related genes was quite expectable, as energy demand and energy usage are dependent on the body temperature, which in fish is dependent on the surrounding temperature. In addition, the formation of creatinine from creatine is temperature dependent. A high temperature increases the formation of creatinine (Lempert 1959), thus withdrawing creatine from the creatine/creatine phosphate pool. Furthermore, creatine kinase activity depends on acclimation temperature in rat (Terblanche et al. 1998), indicating changes in the creatine system.

## Conclusion

In summary, we firstly identified the open reading frames of the creatine-related genes *GATM*, *GAMT*, *CKM* as well as a fragment of *SLC6A8* in rainbow trout. Differences in their gene expression between BORN and import rainbow trout may be due to or may contribute to the so far found differences between both strains. Furthermore, differences in their gene-expression regarding acclimation temperatures indicate a regulation of creatine synthesis and usage under different temperatures. However, rainbow trout of both strains showed a tissue- and temperature-dependent expression pattern that was clearly different from the patterns described in mammals and other teleost’s so far. In rainbow trout not only creatine usage seems to take place in the muscle but also a big part of creatine synthesis.

## Electronic supplementary material

Additional file 1: **Multiple sequence alignments of protein sequences of creatine-related enzymes.** Protein sequences of GATM (a), GAMT (b) and CKM (c) from *Oncorhynchus mykiss* (*Om*), *Salmo salar* (*Ss*), *Takifugu rubripes* (*Tr*), *Danio rerio* (*Dr*), *Ictalurus punctatus* (*Ip*), *Oryzias latipes* (*Ol*), *Homo sapiens* (*Hs*), and *Branchiostoma belcheri tsingtauense* (*Bb*) were aligned to each other. The rulers give positions of the alignment. Conserved, identical amino acids are shaded black, similar ones grey. S marks amino acids of S-adenosylmethionine binding sites, A ADP binding sites, C creatine binding sites, and L predicted members of the substrate specificity loop. (TIFF 2 MB)
