# Supplementary figures and images for: Creatine metabolism differs between mammals and rainbow trout (Oncorhynchus mykiss)
Source: Springerplus. 2014 Sep 9;3:510. doi: 10.1186/2193-1801-3-510 (PMC4167887; doi:10.1186/2193-1801-3-510)

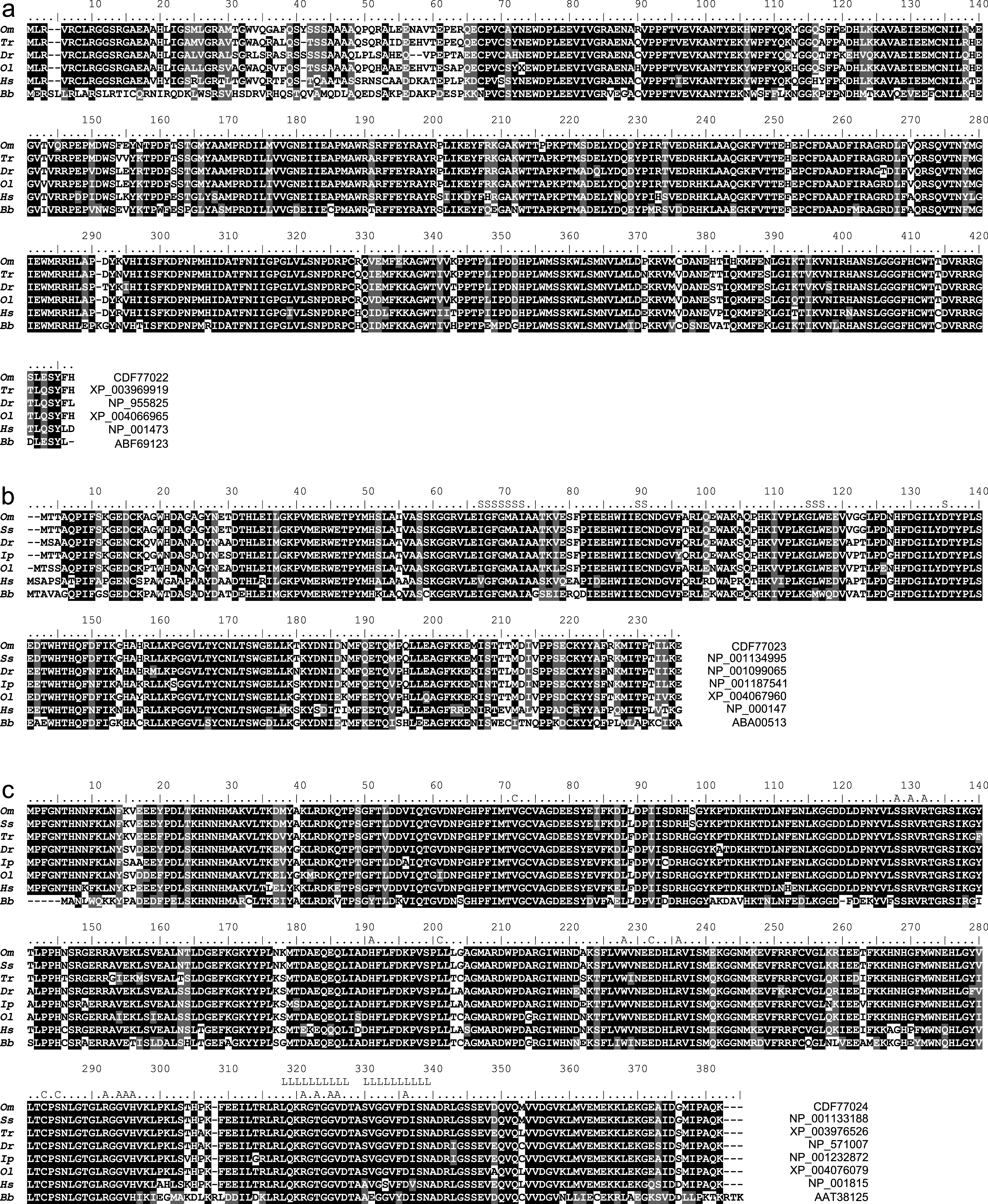

Supplement: Supplementary file 1 — Additional file 1: Multiple sequence alignments of protein sequences of creatine-related enzymes. Protein sequences of GATM (a), GAMT (b) and CKM (c) from Oncorhynchus mykiss (Om), Salmo salar (Ss), Takifugu rubripes (Tr), Danio rerio (Dr), Ictalurus punctatus (Ip), Oryzias latipes (Ol), Homo sapiens (Hs), and Branchiostoma belcheri tsingtauense (Bb) were aligned to each other. The rulers give positions of the alignment. Conserved, identical amino acids are shaded black, similar ones grey. S marks amino acids of S-adenosylmethionine binding sites, A ADP binding sites, C creatine binding sites, and L predicted members of the substrate specificity loop. (TIFF 2 MB) [file 40064_2014_1213_MOESM1_ESM.tiff]
